# Epigenetic bystander-like effects of stroke in somatic organs

**DOI:** 10.18632/aging.100447

**Published:** 2012-03-31

**Authors:** Anna Kovalchuk, Michael Lowings, Rocio Rodriguez-Juarez, Arif Muhammad, Slava Ilnytskyy, Bryan Kolb, Olga Kovalchuk

**Affiliations:** ^1^ Department of Biology, University of Lethbridge, Lethbridge, Alberta, T1K 3M4; ^2^ Department of Neuroscience, University of Lethbridge, Lethbridge, Alberta, T1K 3M4

**Keywords:** disease, stroke, methylation, acetylation

## Abstract

Clinical evidence suggests that stroke may lead to damage of somatic organs. This communication of damage is well-established in the case of exposure to genotoxic agents is termed a bystander effect. Genotoxic stress-induced bystander effects are epigenetically mediated. Here we investigated whether stroke causes epigenetic bystander-like effects in the liver, kidney and heart. We found a significant increase in the levels of H3K3 acetylation and H3K4 trimethylation, as well as a decrease in the H3K9 trimethylation in the kidney tissue of stroked rats. Furthermore, here we for the first time show changes in the gene and microRNA expression profile in the kidney tissues of stroked rats, as compared to intact control animals. Interestingly, the observed changes were somewhat similar to those reported earlier in kidney injury, inflammation, and acute renal failure. Our data explain the recent epidemiological evidence for the increased incidence of acute kidney injury post-stroke and provide an important roadmap for the future analysis of the mechanisms and cellular repercussions of the stroke-induced bystander-like effects in distal somatic organs.

## INTRODUCTION

Ischemic stroke results from the occlusion of an afferent blood vessel and the subsequent reduction of the blood and oxygen supply to the affected brain regions. It accounts for roughly 80% of all clinical strokes [1]. Overall, oxidative stress and cell death (apoptosis and necrosis) are the key molecular processes that are involved in ischemic stroke.

Interestingly, exposure to genotoxic stressors such as toxic chemicals or radiation also causes oxidative stress and apoptosis [2-3]. Indeed, it has been long-established that naïve cells that have been in direct contact with genotoxic stress-exposed cells or have received a certain distress signal from exposed cells exhibit signs of genome destabilization [4]. Such an exposure communication process is termed a *bystander effect*. This effect is well-documented in cells *in vitro* upon radiation exposure. Recent studies have also shown that chemotherapy can lead to bystander effects in the neighboring cells [5].The bystander effect also manifests itself *in vivo* in the tissue and whole-organism context. It was recently shown that cranial irradiation leads to a wide variety of cellular effects in the distal unexposed spleens, testes, and livers of laboratory animals [6-7].

Does ischemic stroke cause a similar bystander-like effect? This question remains to be answered. We hypothesized that ischemic stroke will affect the entire organism and will result in bystander-like effects in the livers, kidneys, and hearts of the animals. We also predicted that, similarly to the irradiation bystander phenomenon [6, 8], the stroke-induced distal bystander-like effects might be epigenetic in nature.

Epigenetic changes are meiotically and mitotically stable alterations in gene expression that are not based on DNA sequence changes and that involve processes that affectchromatin structure, such as DNA methylation, histone modifications, and small RNA-induced silencing [9]. Cytosine DNA methylation is the most extensively studied epigenetic process[10]. It is known to be associated with an inactive chromatin state and repressedgene expression [11-12]. In mammals, three DNA methyltransferases (DNMT1, DNMT3a, and DNMT3b) partake in establishing (DNMT3a and DNMT3b) and maintaining (DNMT1) DNA methylation patterns [13-14]. Proper maintenance of DNA methylation is important for maintaining genome stability, and altered DNA methylation has been well documented in cancer, immunological, cardiovascular, developmental, neurological and psychiatric disorder and aging [15-17].

Changes in DNA methylation do not appear to be isolated, independent events; they often accompany chemical modifications to histone proteins[12, 18-19]. Numerous histone modifications include (not exclusively) acetylation, methylation, and phosphorylation. Histone acetylation leads to more relaxed chromatin packaging and increased gene expression. Histone deacetylation has an opposite effect. Histone methylation is not as straightforward and may lead to both chromatin compaction and relaxation depending upon the residue that is modified [20-21].

Furthermore, classical, genotoxic stress-induced bystander effects are mediated in part through small RNAs — specifically, microRNAs [22]. MicroRNAs are small, single-stranded non-coding RNAs that regulate gene expression at the post-transcriptional level [23]. To control translation of target mRNAs, microRNAs associate with RNA-induced silencing complex (RISC) proteins and bind to the 3' UTR of mRNAs, thus serving as translational suppressors and thereby regulating the production of proteins and affecting many cellular functions, including proliferation, differentiation, cell death, senescence and others [24, 25].

Here, we analyzed epigenetic changes in the liver, kidney, and heart tissue of control and stroked animals. These organs were chosen based on some of the scarce, relevant clinical reports suggesting the indirect impact of stroke on their function in affected patients.

## RESULTS AND DISCUSSION

### Lack of DNA methylation changes in liver, kidney, and heart tissue of stroked animals

**Figure 1:**
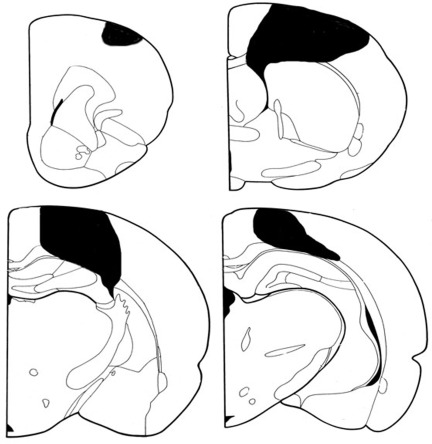
Coronal sections representing the extent of a typical stroke Though this was a somewhat surprising finding, we note that the cytosine extension assay measures global net changes in DNA methylation and does not provide us with the locus-specific DNA methylation details. Thus, we cannot exclude the possibility that there still may be loci that have lost or gained DNA methylation in the tissues of stroked animals. Indeed, recently it has been shown that alterations in DNA methylation in cancer cells occur in defined regions, suggesting localized, and not random, global deregulation of DNA methylation [27-28]. A detailed analysis of locus-specific DNA methylation changes may be needed to investigate the precise gene and locus-specific nature of DNA methylation changes.

**Figure 2:**
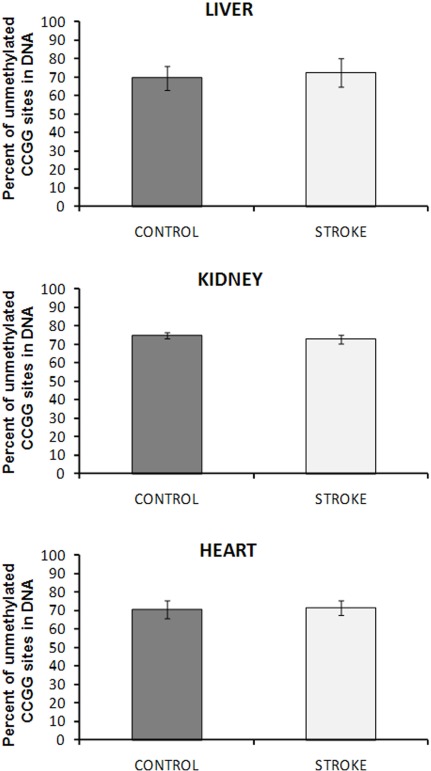
DNA methylation in liver, heart and kidney tissues of control and stroked rats The absolute percent of double-stranded unmethylated CCGG sites was calculated by relating the data of HpaII and MspI digests. Data are presented as mean values ± SD.

The focal, permanent devascularization (stroke) has been described in detail elsewhere [26]. Figure 1 provides a schematic description of the stroke model used in our study. To test if changes in global DNA methylation wereobserved in liver, kidney and heart tissues of control and stroked animals, we employed the well-established *Hpa*II-based cytosine extension assay. This assay measures the proportion of unmethylated CCGG sites in genomic DNA [8]. We found that all tissues of the control and stroked animals had comparable DNA methylation levels (Figure 2).

### Subtle changes in the levels of DNA methyltransferases and methyl-binding protein MeCP2 in the distal somatic tissues of stroked animals

**Figure 3:**
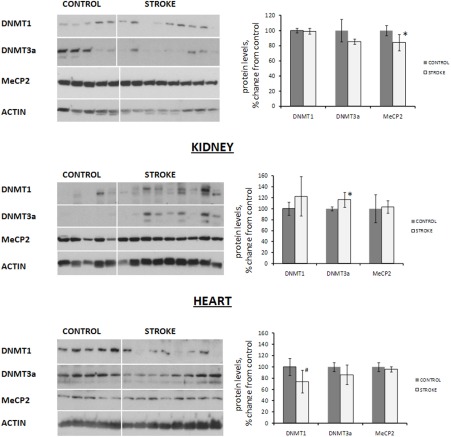
Expression of DNA methyltransferases and methyl-binding protein MeCP2 in liver, heart and kidney tissues of control and stroked rats Protein levels relative to those of control animals are shown as the mean ± SD, ±significant, p < 0.05, Student's t-test. Representative blots from among three independent repeats are shown.

Analysis of the levels of DNMT1 and DNMT3a the liver, kidney, and heart tissues of control and stroked animals revealed interesting and tissue-specific protein expression changes. We noted that DNMT1 levels were subtly (p<0.10) decreased in the heart tissue of stroked animals as compared to controls. In the kidneys of stroked animals, contrarily, the levels of DNMT3a were slightly increased (p<0.05). In the liver tissue of stroked animals, both DNMT1 and DNMT3a levels remained unchanged (Figure 3).

In addition to altered levels of DNMTs, we noted a subtle change in the levels of methyl-binding protein MeCP2 in the liver tissue of stroked rats (Figure 3).

This protein was shown to interact with methylated DNA and affect the methylation-mediated chromatin remodeling and gene silencing [29]. The contributions of DNMTs and MeCP2 to bystander-like effects induced by stroke in distal somatic organs have yet to be analyzed, as those may contribute to establishing some locus-specific DNA methylation patterns.

### Histone methylation and acetylation changes in the distal tissues of stroked animals

Changes in histone modifications directly affect chromatin packaging and gene expression [30]. Among numerous histone modifications, trimethylation of histone H3 lysine 9 (H3K9) is associated with chromatin compaction and decreased expression [31]. Acetylation of H3K3, contrarily, leads to increased expression and chromatin relaxation [30]. These modifications may be mutually exclusive. Furthermore, trimethylation of lysine 4 on histone H3 (H3K4) is also known to correlate with increased expression and relaxed chromatin.

We analyzed the levels of these H3K9 and H3K4 modifications using western immunoblotting. While no changes were seen in the liver, and only one modification occurred in the heart, the changes in the kidney tissues of stroked animals were the most pronounced.

**Figure 4:**
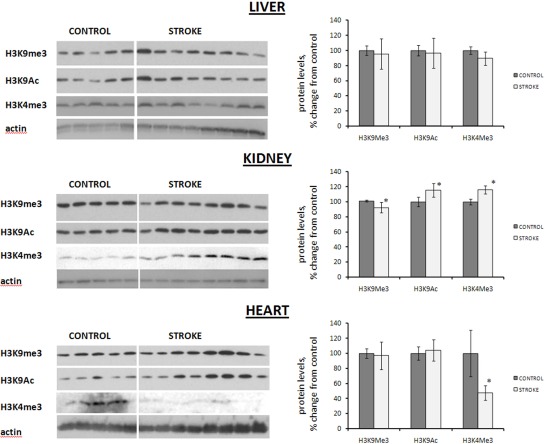
Histone modification levels in liver, heart and kidney tissues of control and stroked rats Levels relative to those of control animals are shown as the mean ± SD. Representative blots from among three independent repeats are shown. ±significant, p < 0.05, Student's t-test.

Indeed, on the one hand, no histone acetylation or methylation changes were seen in the liver tissue of stroked rats, and in the heart tissue of stroked animals, we noted a significant decrease in the level of trimethylated H3K4. On the other hand, we noted a statistically significant 15% increase in the levels of acetylated H3K9, and a corresponding decrease in methylated H3K9, in the kidney tissue of stroked animals as compared to control animals (Figure 4). Likewise, we noted a significant 16% increase in the levels of H3K4 trimethylation in the kidney tissue of stroked rats compared to the controls (Figure 4).

Interestingly, H3K4 and H3K9 methylation levels are mutually exclusive, and this relationship is conserved from yeast to humans. Recent findings show that increased H3K4 trimethylation plays an important part in the renal inflammatory response and is associated with proximal tubal injury, acute renal failure, and ischemic renal injury. On the other hand, decreased levels of H3K4 trimethylation were reported to be a negative prognostic indicator for renal cell carcinoma. Moreover, recent studies show that the acetylation of histone H3K9 and loss of H3K9 trimethylation may be important in progressive glomerulosclerosis [32-33]. Stroke-induced effects in the kidney may in turn be relatively similar to those induced by hemorrhage and glomerulosclerosis.

In sum, the observed histone changes may be indicative of the alteration in chromatin structure and gene expression in the kidneys of stroked rats. These new intriguing findings deserve special attention in the future. It is especially important to discern the locus specificity of the observed global histone changes and any relationship between the observed changes and inflammation.

### Altered gene expression in the distal kidney tissues of stroked animals

Decreased levels of methylated H3K9, increased levels of acetylated H3K9, and methylated H3K4 may in turn lead to the activation of gene expression. Therefore we conducted an analysis of differential gene expression inthe kidney tissue of control and stroked rats. Our analysis revealed that 22 genes were differentially expressed in the kidney tissues of stroked rats as compared to the controls. Interestingly, all of these genes were up-regulated. The expression of four randomly selected genes—Cml3, Rps27a, RGD1562073, and Tnfsf13—was verified by qRT-PCR (Table 1).

**Table 1:** 

Gene ID	Gene name	Cellular function/process	Fold induction (array)	Fold induction (qRT-PCR)	Condition/disease association
Alad	δ-aminolevulinic acid dehydrase	heme biosynthesis	1.3		kidney response to toxic compounds
Cln6_predicted	ceroid-lipofuscinosis, neuronal 6		1.3		NCL
Cml3	camello-like 3, transcription factor	transcription	1.6	1.9	
Degs1	degenerative spermatocyte homolog 1, lipid desaturase	membrane fatty acid desaturase	1.4		cancer, metastasis
Exosc7	exosome component 7	exosome function	1.3		
Krt2-7	a member of the type I (acidic) keratin family	cytoskeleton	1.5		nephropathy
LOC289715	Rattus norvegicus similar to ribosomal protein L37	translation	1.3		cancer
Mrps36_predicted	mammalian mitochondrial ribosomal protein	translation	1.3		
Plac8_predicted	Rattus norvegicus placenta-specific 8 (predicted)		1.6		
RGD1310925_predicted	small nuclear ribonucleoprotein 27 (U4/U6.U5)	splicing	1.3		
RGD1559951_predicted	similar to 60S ribosomal protein L37a	translation	1.4		cancer, DNA damage response
RGD1559960_predicted	similar to sulfotransferase K1		1.9		
RGD1561195_predicted	similar to ribosomal protein L31	translation	1.3		rat cerebral ischemia
RGD1561310_predicted	similar to ribosomal protein L37	translation	1.4		cancer
RGD1562073_predicted	similar to ribosomal protein S17	translation	1.9	1.6	
RGD1563547_predicted	hypothetical protein LOC360478		1.4		
Rpl39	60S ribosomal protein L39	translation	1.5		
Rps27a	ribosomal protein S27a	translation	1.4	1.5	
Tnfsf13	tumor necrosis factor ligand superfamily member 13	ligand, TNF ligand family	1.3	1.3	cancer, lupus nephritis
Umps	uridine 5'-monophosphate synthase	UMP synthesis	1.4		

Among the upregulated genes, eight encode ribosomal proteins. The products of ribosomal protein genes—ribosomal proteins—are involved in translation and constitute integral parts of ribosomes [34]. The small 40S ribosomal subunit contains approximately 32 ribosomal proteins (RPS proteins), while the large 60S ribosomal subunit harbors approximately 47 ribosomal proteins (RPL proteins) [34]. Ribosomal proteins are crucial for normal cellular functioning, yet not much is known about their roles in various cellular processes. Recent reports show that the increased expression of ribosomal proteins has also been associated with increased cellular proliferation and growth. Furthermore, the increased expression of ribosomal proteins, such as RPS8, RPL12, RPL23A, RPL27, RPL37, and RPL30, has been found in various different tumor types [34-35]. Furthermore, RPS27 protein partakes in the genotoxic stress response [36-37]. Notwithstanding this, it is unclear if these changes in ribosomal protein expression are causally related to tumorigenesis. In our study, we found a significant increase in the expression of genes encoding for ribosomal proteins. The cellular and organismal reper-cussions of these changes need to be analyzed further.

Another gene that we found to be significantly upregulated in the kidneys of stroked animals was aminolevulinate dehydratase (ALAD)[38]. ALAD is one of the key heme biosynthesis enzymes. This enzyme is important for normal kidney function and is involved in kidney responses to heavy metals and other toxicants [39]. In our study, ALAD expression was 1.3 times higher in the kidneys of stroked rats as compared to controls. The role of ALAD in post-stroke acute kidney injuries needs to be further analyzed.

We also noted an upregulation of the Tnfsf13 gene, also known as APRIL (a proliferation-inducing ligand). Tnfsf13/APRIL belongs to the tumor necrosis factor superfamily and is important for B-cell development, maturation, and survival. It contributes to human autoimmune diseases and cancer. Furthermore, recent studies have shown that Tnfsf13/APRIL is significantly upregalated in the glomeruli and tubulointerstitium in the course of human proliferative lupus nephritis [40-41].

Overall, based on the literature and database analysis, many genes that were upregulated in the kidney tissue of the stroked rats were previously shown to be associated with various neoplasms, including urinary neoplasms, kidney inflammation, and kidney responses to toxic compounds (Table 1).

### Altered expression of microRNAs in kidney tissue of stroked rats

**Figure 5:**
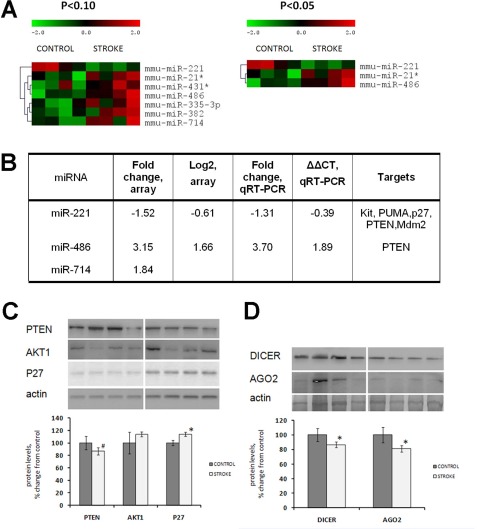
Altered expression of miRNAs, microRNA target proteins and microRNAome machinery in the kidney tissue of control and stroked rats **(A)** Hierarchical clusters of differentially expressed miRNA genes in the kidney tissue of control and stroked rats (as determined by *t*-test). **(B)** Fold changes of miRNA expression as measured by both microarray and qRT-PCR. **(C)** Levels of miRNA targets PTEN, AKT1 and P27. **(D)** Levels of miRNA processing enzymes DICER and AGO2. Protein levels relative to those of control animals are shown as the means ± SD, ±significant, p < 0.05, Student's t-test. Representative western blots from among three technical repeats are shown.

Altered levels of histone modifications may also affect expression of microRNAs. Therefore, next we analyzed microRNA expression in the kidneys of control and stroked animals, using microRNA microarrays. Cluster analysis revealed that the kidney tissue of stroked animals was characterized by altered microRNA expression. Specifically, we noted a significant decrease in the levels of miR-221 and a significant increase in the levels of miR-486 and miR-714 (p<0.05) (Figure 5A). Altered expression of miR-221 and miR-486 was independently confirmed by quantitative real-time PCR analysis (Figure 5B).

MicroRNAs that exhibited altered expression in the kidney tissue of stroked rats are known to regulate a variety of cellular processes by targeting important proteins. miR-221 was shown to target and control expression of several key proteins such as p27 [42-43], phosphatase, tensin homolog (PTEN) [44], Kit oncogene, and others. The most important targets of miR-486 are PTEN and Foxo1a, which negatively affect phosphoinositide-3-kinase (PI3K)/Akt signaling [45].

We noted that levels of p27 were significantly elevated in the kidneys of the stroked rats (Figure 5C). Interestingly, we saw a subtle (p<0.10) decrease in the level of PTEN. This protein is targeted by both miR-221 and miR-486, which exhibit opposite expression tendencies: miR-221 is down-regulated and miR-486 is unregulated in the kidney tissue of stroked animals compared with the control ones. Since PTEN is a target of both of these microRNAs, and miR-221 was 1.5-fold down-regulated, and miR-486 was 3.15-fold up-regulated, PTEN expression in the stroked kidneys was slightly down-regulated as compared to the control kidneys.

The observed decrease of the PTEN level in the kidneys of stroked rats is interesting because it was recently shown that PTEN downregulation may contribute to renal cell hypertrophy and matrix expansion. Also, a decrease of PTEN and elevated levels have been reported in diabetes kidney injury [46]. Recent studies have also reported increased levels of p27 and concurrent activation of Akt signaling in renal cell carcinoma [47].

Having seen some deregulation of microRNA expression in the kidneys of stroked animals, we next analyzed the levels of the microRNA-processing machinery proteins Dicer and Argonaute 2 (Ago2) in the kidney tissue of control and stroked rats. Our analysis revealed a slightly decreased level of both Dicer and Ago2 in the kidneys of stroked rats (Figure 5D). Dicer, a key enzyme for microRNA production, plays important roles in the kidneys. Mice that lacked Dicer in the proximal kidney tubular cells exhibited normal renal function and histology despite a global down-regulation of microRNAs in the renal cortex [48]. Furthermore, these mice were very resistant to renal ischemia-reperfusion injury. They exhibited better renal function, less tissue damage, lower tubular apoptosis, and improved survival as compared to the wild-type animals with normal Dicer levels [48]. Overall, Dicer is very important for kidney function, especially for the juxtaglomerular apparatus [49]. Dicer and microRNAs play important roles in kidney disease [50-51]. However, the role of Dicer and Ago2 in stroke-induced bystander-like responses in the kidneys need to be further substantiated.

## CONCLUSIONS AND OUTLOOK

Animals that suffered from ischemic stroke exhibited molecular epigenetic changes in the distal kidney, as well as in heart and liver, tissues. Interestingly, the aforementioned epigenetic changes were the most pronounced in the kidney tissue. The most striking changes were seen in the levels of H3K4 methylation, H3K3 methylation, and acetylation in the kidneys of stroked animals. Additionally, we also noted intriguing gene and microRNA expression changes in the kidney tissues of stroked rats. The sensitivity of kidney tissue to the distal effects of stroke is an important novel finding.

Recent data clearly showed that kidney damage and acute kidney failure is one of the very common, yet overlooked, post-stroke complications [52-53]. Epidemiological evidence suggests that acute kidney damage contributes significantly to post-stroke mortality, yet the mechanisms of post-stroke kidney damage have not been completely understood [53]. Our animal model-based data are the first to show the existence of altered levels of histone modification in the kidney tissues of stroked animals.

These epigenetic changes may significantly affect gene expression and chromatin organization in the kidney cells. Even though the observed epigenetic changes were seemingly subtle, even subtle epigenetic changes may often lead to altered gene expression and altered genome stability. The mechanisms of the occurrence, locus specificity, and cellular repercussions of stroke-induced bystander effect-like epigenetic changes need to be further analyzed. Our study serves as a roadmap for the future analysis of the stroke-induced bystander-like effects in distal tissues and organs.

## MATERIALS AND METHODS

### Animal model

Male Long-Evans rats (N= 16, 120-150 days old) were used as subjects. Eight rats received a motor cortex stroke and eight rats served as normal controls.

*Surgery*: Under isofluorane anaesthesia, a rectangular hole was drilled into the frontal and parietal bones running from + 3 to -4mm anterior/posterior to the Bregma and running laterally from 1.5 to 4.5mm from midline. The focal, permanent devascularization (stroke) has been described in detail elsewhere [26]. Specifically, the dura was removed and a sterile saline-soaked cotton swab was used to wipe the pia and attached blood vessels from the cortical surface (Figure 1). Surgery was performed on the hemisphere contralateral to the preferred paw in a skilled reaching test (data not shown) and was roughly equally divided between the left and right hemispheres. There was no mortality after surgeries; however, for 24 h after the devascularization animals were lethargic and sensitive to touch, after which they returned to their pre-surgical behaviour. Animals were humanely euthanized and kidney, liver and heart were harvested and immediately frozen for analysis.

### Western immunoblotting analysis

Western immuno-blotting was conducted on tissue lysates as described before [8] using antibodies against DNMT1 (1:1000; Santa Cruz Biotechnology) MeCP2, DNMT3a, ac-H3K9, me3-H3K9, me3-H3K4, Dicer, Ago2 (1:1000; Abcam), PTEN, p27, Akt1 (1:1000; Cell Signalling). The protein levels were related to those in controls. Two or three technical replicates were used for each immunoblotting.

### DNA methylation analysis

Genomic DNA was isolated from tissues, and the cytosine extension assay for determination of the absolute percent of double-stranded unmethylated CCGG sites were conducted as described previously [8].

### microRNA expression analysis

MicroRNA microarray analysis was conducted using kidney tissue of control and stroked rats. Total RNA was extracted using TRIzol Reagent (Invitrogen, Burlington, ON). Microarray analysis was performed by LC Sciences (Houston, TX) as described before [8].

### Quantitative real-time PCR (qRT-PCR) expression enalysis

qRT-PCRs were performed on samples from all groups using TaqMan Primer sets specific for miR-486 and miR-221. SnoRNA202 served as a control.

### Statistical tests

Results are presented as mean±S.D. Statistical analyses were conducted by the Student`s *t*-test.
